# The role of SGK1 in neurologic diseases: A friend or foe?

**DOI:** 10.1016/j.ibneur.2024.12.003

**Published:** 2024-12-06

**Authors:** Xiuze Chen, Haixian Kang, Yechen Xiao

**Affiliations:** a Department of Biotechnology, Basic Medical School, Guangdong Medical University, Dongguan 523808, China; b Shunde Women and Children's Hospital of Guangdong Medical University, Foshan 528300, China

**Keywords:** Serum and glucocorticoid-regulated kinase 1, Neurological disorders, Alzheimer's disease, Parkinson's disease

## Abstract

Serum and glucocorticoid-regulated kinase 1 (SGK1), a member of the AGC family of serine/threonine protein kinases, is one of the most conserved protein kinases in eukaryotic evolution. SGK1 is expressed to varying degrees in various types of cells throughout the body, and plays an important role in hypertension, ion channels, oxidative stress, neurological disorders, and cardiovascular regulation. In recent years, a number of scholars have devoted themselves to the study of the role and function of SGK1 in neurological diseases. Therefore, this article reviews the role of SGK1 in Alzheimer's disease, Parkinson's disease, epilepsy, stroke and other neurological diseases in recent years, and puts forward some insights on the role of SGK1 in neurological diseases and its relationship with disease activities.

## 1 Introduction

Serum and glucocorticoid-inducible kinase (SGK) was initially isolated in a rat mammary tumor cell line in 1993. When serum and glucocorticoids stimulate the cells, the mRNA content of SGK can be rapidly upregulated within 30 min, so it was named serum and glucocorticoid-regulated kinase(Lang and Cohen, 2001). SGK belongs to the AGC family of serine/threonine protein kinases (protein kinases A, G, and C) and has three different isoforms, SGK1, SGK2, and SGK3 (Kobayashi et al., 1999). The SGK1 gene is localized on chromosome 6q23 (Lang et al., 2006). Glucocorticoids are the main stimulus for the transcriptional response of SGK1 because the promoter of SGK1 contains a sequence shared by the glucocorticoid-responsive element 1.0 kb upstream of the transcriptional start(Webster et al.*,* 1993). The gene of SGK1 has a total length of 2.4 kb and encodes a protein with a relative molecular mass of 49 KD. SGK1 is structurally composed of an N-terminal (amino-terminal), intermediate catalytic domain, and C-terminal (carboxy-terminal) (Zhao et al., 2007). The primary form of SGK1 is inactive, so it needs to be activated by phosphorylation.The two phosphorylation sites are located in Thr256 above the activation loop of the intermediate catalytic domain, and Ser422 at the C-terminal end of the SGK1 (Pearce et al., 2010). Although SGK1 is a conserved protein kinase, its high expression level and wide distribution in various organs make it play an important role in the regulation of life activities.

Neurological diseases encompass a wide variety of diseases, including Parkinson's disease, Alzheimer's disease and stroke, etc. In terms of causes of death, neurological diseases are second only to cardiovascular diseases. In 2015, 16.8 % of global deaths were caused by neurological disorders (Group, 2017). Particularly in low- and middle-income countries, the number of deaths from neurological diseases is expected to increase further globally due to population growth and aging (Feigin et al., 2020). Although many countries have invested heavily in tackling this challenge, few therapeutic agents have been approved (Alessandrini et al., 2019), because the causative mechanisms of many neurological disorders have yet to be fully investigated. In the face of these urgent problems, many scholars found that SGK1 plays a significant role in the occurrence and development of Parkinson's disease, stroke, etc., suggesting that the regulation of SGK1 may be a therapeutic target or a potential drug. Therefore, in this paper, we briefly review the progress of research on SGK1 in some neurological diseases.

## 2 Generation and regulation of SGK1

**Fig. 1:**
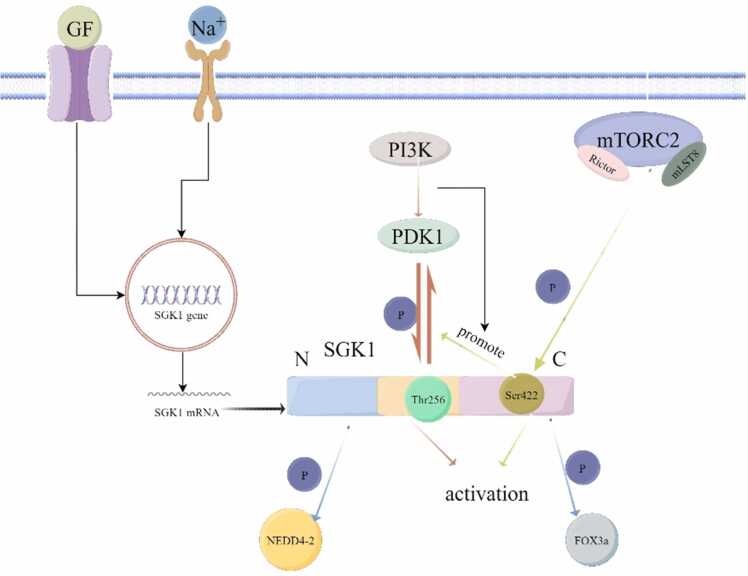
Expression and regulation of SGK1. The transcription of SGK1 mRNA is regulated by extracellular signals (e.g., GF, sodium ions, etc.). Activation of SGK1 is mediated by activation of Thr256 phosphorylated by PI3K/PDK1. In addition, mTORC2 can also phosphorylate SGK1 at Ser422, and phosphorylation of Ser422 also promotes the activation of Thr256.

The production of SGK1 in the human body is regulated by transcriptional and post-translational regulation. Many cytokines such as hormones, transforming growth factor, interleukin 6, and gonadotropin can rapidly regulate SGK1 expression in the transcription level (Lang et al., 2009). SGK1 is also regulated by post-translational phosphorylation/dephosphorylation modifications. As mentioned earlier, there are two main phosphorylation sites for SGK1 activation and production, one is Thr256 in the intermediate structural domain, and the other one is located at the Ser422 of C-terminus. Thr256 is mainly activated by the phosphatidylinosine-3- kinase (PI3K) pathway, and also participates in the signal transduction pathway of PI3K after activation (Lee et al., 2006). And Ser422 is mainly activated by phosphorylation of Rictor and mLST8, which are the subunits of the rapamycin receptor 2 (mTORC2) (García-Martínez and Alessi, 2008). Interestingly, mTORC2 activates Ser422 of SGK1, triggering SGK1 to interact with PDK1 in the PI3K pathway, which further activates SGK1 through phosphorylation at Thr256 (Firestone et al., 2003). Thus, SGK1 is ultimately phosphorylated by PDK1 and mTORC2, which activates the enzymatic function of SGK1 (Fig. 1).

Activated SGK1 is involved in many signaling pathways that regulate human life activities. As a serine/threonine kinase, SGK1 catalyzes the phosphorylation of many target proteins, including NDRG1, FOXO3a, NEDD4–2, TSC2, ULK1, and β-catenin (Cicenas et al., 2022). SGK1 plays an important role in the PI3K-AKT signaling pathway. Inhibition of AKT and phosphorylated glycogen synthase kinase 3β (GSK3β) increases apoptosis, but PI3K/AKT-mediated up-regulation of SGK1 reverses this trend and prevents apoptosis (Cong et al., 2014; Zhang et al.*,* 2014), which has a positive effect on cerebral ischemia, Alzheimer's disease, and other neurological diseases. When PI3K is negatively regulated, phosphorylation of AKT results in downstream phosphorylation of IκB, activation and transport of NF-κB into the nucleus, thus mediating a series of inflammatory conditions, which further impacts on neurological disorders (Lang and Voelkl, 2013).

## 3 The role and influence of SGK1 on neuropathic diseases

**Fig. 2:**
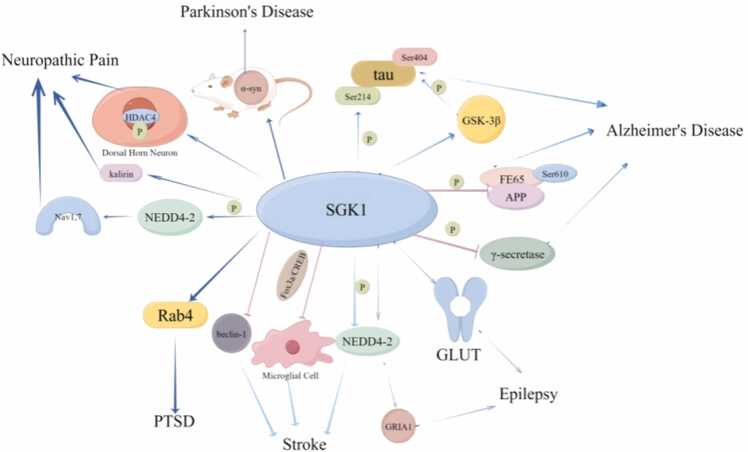
Signaling pathways and target genes mediated by SGK1 in some neurological diseases. In different neurological diseases, SGK1 activates different downstream signaling molecules through multiple regulatory modes such as phosphorylation, which plays a positive or negative role.

SGK1 is involved in regulating and repairing the physiological functions of the nervous system (Fig. 2). On the one hand, SGK1 can maintain brain functional connectivity, promote neuronal survival and information transmission, brain energy metabolism, and regulate the normal transport of sodium ions in nerve cells to improve learning and memory disorders(Xing et al., 2023). On the other hand, SGK1 promotes the release of excitatory transmitters from the central system, and thus it has some neurological damaging effects (Matschke et al., 2015). Therefore, Clarifying whether SGK1's role in a particular disease is positive or negative will help us further understand and treat neurological diseases through SGK1 as a therapeutic target.

### 3.1 SGK1 and Alzheimer's disease

Alzheimer's disease (AD) is the most common type of dementia and is defined as a slowly progressive neurodegenerative disease characterized by neuroinflammatory plaques and neurofibril tangles (nfts), which are the result of accumulation of β-amyloid (Aβ) and hyperphosphorylation of tau proteins consisting of nfts in the brain's medial temporal lobes and neocortical structures (De-Paula et al., 2012). Therefore, in order to treat AD, we can start by reducing the production of Aβ and the hyperphosphorylation of tau protein. Aβ is produced by β-secretase (β-site amyloid precursor protein cleavage enzyme 1) and γ-secretase by hydrolyzing and processing the amyloid precursor protein (APP) (Cai et al., 2018; Chin-Chan et al., 2018), and SGK1 seems to play an important role in inhibiting APP production.

At the cellular experimental level, SGK1 was found to down-regulate the stability of the γ-secretase component nicastrin protein through the mechanism of proteasomal and lysosomal pathways, thereby inhibiting γ-secretase activity and further down-regulatingγ-secretase-dependent APP cleavage (Mo et al., 2012). By comparing wild-type and SGK1-deficient MEF cells, γ-secretase was found to be higher in deficient cells than in wild-type cells, and overexpression of SGK1 in SGK1-deficient cells led to γ-secretase-dependent transactivation(Mo et al., 2012). In another pathway, a brain-enriched articulin FE65, was found to promote the amyloidogenic process of APP by preventing the degradation of APP holoproteins, but SGK1 can phosphorylate FE65 on Ser610, and this phosphorylation attenuates FE65 binding to APP, thus inhibiting the amyloidogenic process of APP (Chow et al., 2015). At the animal experimental level, by over-expressing SGK1 in the hippocampus of a female middle-aged APP/ PS1 mouse model, it was found that SGK1 over-expression significantly increased the level of hippocampal IDE (enzyme for degrading Aβ), which significantly reversed the spatial memory impairment caused by AD, and at the same time, SGK1 overexpression promotes the increase of non-amyloid metabolism and degradation of Aβ induced by APP, leading to a decrease in the production and deposition of Aβ (Lian et al., 2020). Thus, SGK1 can inhibit Aβ production from both phosphorylating γ-secretase and down-regulating APP protein synthesis.

Meanwhile, some studies have shown that sleep deprivation increases the production of Aβ in the brain (Slats et al., 2013), and by overlapping differentially expressed genes between sleep deprived mice and AD mice, the Sgk1 gene was found to be deregulated in both sleep deprived mice and AD mice, which implies a possible role of SGK1 in the treatment of AD(Wei, 2020). In addition, the development of AD is closely related to changes in the hippocampus, and it has been shown that SGK1 is involved in the regulation of hippocampal synaptic plasticity, spatial learning, and memory formation (Tyan et al., 2008; Yang et al., 2011). SGK1 enhances memory by upregulating the hydrochloride receptor GluR6, and thereby enhancing neuronal excitability (Lang and Shumilina, 2013; Lang et al., 2014). Izumi H et al. found that SGK1 is associated with inhibition of Aβ deposition and improved cognitive function, and plays an important role in hippocampal neuronal plasticity and spatial memory (Izumi et al., 2018). Mengying Liu et al. identified Sgk1 in the hippocampus as a novel target gene mediating ovarian estrogen regulation of spatial memory, synaptic plasticity, and Aβ accumulation by transcriptomics analysis (Liu et al., 2022). Moreover, glucocorticoids modulate the expression of glutamatergic α-amino-3-hydroxy-5-methyl-4-isoxazolepropionic acid (AMPA) and n-methyl-d-aspartate (NMDA) receptors through the action of SGK1, affecting hippocampal learning ability and synaptic plasticity (Lengel et al., 2022). One study also found enhanced SGK1 activity in patients with severe AD, which protects the brain to inhibit apoptosis by phosphorylating NDRG1 and FOXO3a (Iqbal et al., 2015; Sahin et al., 2013).

However, parsing AD in terms of nfts composed of hyperphosphorylated tau proteins, SGK1 has a different role. Histone methylation (H3K4me3) was found to be elevated in the prefrontal cortex (PFC) of the P301S Tau mouse model, and SGK1 has been shown to be one of the regulatory target genes for elevated H3K4me3. Promoting the reduction of H3K4me3 by inhibiting the expression of Sgk1 contributed to the significant reduction of hyperphosphorylated tau proteins in the frontal cortex of the tau transgenic mice (Cao et al., 2020). At the same time, SGK1 activity in mice was inhibited by SGK1 inhibitor GSK650394, and it was found that this treatment promoted the recovery of normal cognitive behavior and synaptic function of PFC in P301S Tau mice, which verified the relationship between SGK1 and AD in positive and negative aspects (Cao et al., 2020). In a mouse model of a high-fat diet, the high-fat diet was found to up-regulate SGK1, leading to increased levels of tau protein phosphorylated at Ser214, which inhibited microtubule-stabilization-associated tau protein activity, as well as to promote SGK1 activation of GSK-3β and induced phosphorylation of tau at the Ser396 and Ser404 loci, thereby promoting tau fibrosis (Elahi et al., 2021). δ-secretase has been found to produce Aβ in the development of AD, which further induces phosphorylation of SGK1 and JAK2, and SGK1 can in turn activate STAT1, and δ-secretase simultaneously cleaves tau proteins and produces the tau(1−368) fragment, which binds to the activated STAT1 and promotes the production of Aβ thereby forming positive feedback regulation with phosphorylated SGK1 (Zhang et al., 2021).

In summary, SGK1 may have a double-edged role for AD, but even though the role played by SGK1 in AD is dichotomous, it is undeniable that SGK1 plays a role in the pathogenesis of AD, and can be used to be an important therapeutic target for the treatment of AD.

### 3.2 SGK1 and Parkinson's disease

Parkinson's disease (PD) is a common neurodegenerative disorder with a global prevalence that has exceeded 6 million (Tolosa et al., 2021). Its pathology is characterized by significant loss of dopaminergic neurons located in the substantia nigra, as well as the formation of intracytoplasmic Lewy bodies and dystrophic neurites (Nguyen et al., 2019).

At the pathophysiologic level, the formation of Lewy bodies is associated with α-synuclein (α-syn) and dopaminergic cell death(Shults, 2006), thus reducing α-syn aggregation and dopaminergic cell death is an important modality in the treatment of PD. In recent years, more and more scholars found that SGK1 expression plays an important role in the aggregation of α-syn. Although the cause of aggregation of α-syn is unknown, it was found that decreased expression of SGK1 in MPTP-induced Parkinson's mouse model and SH-SY5Y cells may lead to dopaminergic cell death through increased expression and aggregation of α-syn (Yeo et al., 2018). In a mouse model of MPTP-induced Parkinson, acupuncture was found to up-regulate the expression of SGK1 thereby decreasing the aggregation of α-syn (Yeo and Lim, 2019). Experiments were also performed using SH-SY5Y cells in which SGK1 was downregulated by siRNA, and it was found that siRNA-induced reduction of SGK1 leads to disruption of dopaminergic cells and an increase of α-syn expression (Yeo and Lim, 2019). Gastrointestinal disorders, as one of the early non-motor symptoms of Parkinson's disease, were found in the colon of a mouse model of MPTP-induced PD, where the decrease of SGK1 expression caused the increase of α-syn and Na-K-atpase α1, while increased Na-K-atpase α1 may reduce the demand for Na/K-pumping atpase β1, leading to a decrease in Na/K-pumping atpase β1, which further contributes to disturbances in the gastrointestinal water balance (Seo et al., 2023). In movement disorders, studies have found that there is also a link between SGK1 and α-syn in the skeletal muscle of MPTP-induced PD mouse models, and MPTP caused a decrease of SGK1 expression, which may lead to an increase of α-syn level in muscle (Heo et al., 2023).

Interestingly, it has been shown that inhibition of SGK1 expression instead facilitates the alleviation of PD symptoms. In glial cells, inhibition of SGK1 was shown to correct the pro-inflammatory properties of glial cells by inhibiting the inflammatory pathways mediated by the intracellular NF-κB signaling pathway, NLRP3-inflammatory vesicles, and the CGAS-STING signaling pathway, which in turn alleviated the symptoms of PD. Moreover, inhibition of SGK1 improves the aggregation of α-syn in glial cells in an *in vitro* model of α-syn pathology (Kwon et al., 2021), further implying that SGK1 inhibition is beneficial for the treatment of PD. However, in terms of the MPTP-induced PD model, SGK1 is drastically upregulated in this model (Stichel et al., 2005), and its elevated expression contributes to the reduction of α-syn aggregation and dopaminergic cell death. Therefore, a deeper exploration of the role of SGK1 in PD may be useful for the treatment of PD.

### 3.3 SGK1 and epilepsy

Epilepsy is a chronic neurological disorder that affects more than 70 million people worldwide. Although more than 20 antiepileptic drugs are available for the symptomatic treatment of epileptic seizures, current medication remains ineffective in about 1/3 of patients with epilepsy (Löscher et al., 2020). The role of SGK1 in epilepsy has also been studied in recent years. A report found that SGK1 mRNA and protein are upregulated after seizures (Wang et al., 2010), specifically they explored the relationship between SGK1 expression and epileptogenesis by measuring SGK1 expression in human brain tissues and experimental models of epilepsy in rats, and found that the expression of SGK1 was enhanced in the temporal lobe neocortex of epileptic patients at different stages of the epileptic process. It is well known that SGK1 and NEDD4–2 are coordinated with each other (Zhou and Snyder, 2005). On the basis of Ji-Eun Kim et al.'s finding that SGK1-NEDD4–2-mediated dysregulation of ubiquitination in GRIA1 may be responsible for refractory seizures (Kim et al., 2021b), it can be speculated that this pathway may be a potential therapeutic target for improving the treatment of intractable epilepsy. They found that ubiquitination of NEDD4–2 is involved in epileptogenesis and that upregulation of SGK1 is associated with the degradation NEDD4–2 in the epileptic hippocampus. Therefore, inhibition of NEDD4–2 degradation could be achieved through downregulation of the expression and content of SGK1, which in turn can regulate epilepsy through the SGK1-NEDD4–2 pathway.

Among genetic epilepsies, Lafora (LD) is a fatal genetic form of myoclonic epilepsy characterized by the presence of dextran inclusion bodies called Lafora vesicles and the absence of laforin and malin in various tissues including muscle, liver and brain (Serratosa et al., 2012). Deficiency of laforin leads to activation of SGK1, which exacerbates plasma membrane-bound glucose transporter protein levels, glucose uptake, and consequent glycogen accumulation, further forming Lafora vesicles (Singh et al., 2013), therefore, inhibition of SGK1 activity may be an effective therapeutic approach. Tuberous sclerosis (TSC) is another type of genetic intractable epilepsy. Inhibition of mTOR pathway signaling (both mTORC1 and mTORC2 are involved) was found to prevent abnormal brain development in TSC during embryogenesis, which in turn prevented TSC from occurring (Tsai et al., 2014)，but inhibition of mTOR will inevitably have an important effect on the activation of SGK1, and thus the role SGK1 in TSC needs to be further explored.

The M current is a neuronal voltage-gated K current formed by tetramerization of Kv7.2 and Kv7.3 subunits that controls resting membrane potential and cellular excitability. The SGK1 subtype, SGK1.1, appears to play an important role in suppressing epilepsy, particularly in neurons where SGK1.1 can specifically activate and up-regulate M currents to reduce the severity and mortality of persistent seizures (Miranda et al., 2013). In Xenopus cells and HEK293 cells with non-inactivated volt-dependent K current expression, SGK1.1 increased the Kv7.2/3 channel current and its membrane abundance without affecting the kinetic characteristics, rapidly introduced K current into the membrane, and thus restored the resting potential more quickly, resulting in reduced sympathetic excitability. Based on the above results, a transgenic mouse model of epilepsy induced by Carthocyanine (KA) further revealed that Nedd4–2 is a substrate for SGK1.1 in hippocampal neurons and constitutes a regulatory pathway by which SGK1.1 regulates M current in the hippocampus (Armas-Capote et al., 2020). Further studies indicated that SGK1.1 interacts with Nedd4–2 in a phosphorylation-dependent manner. Loss-of-function mutant Kv7.2/3 channels fully activated SGK1.1 and partially restored its function, producing partial M currents (Martin-Batista et al., 2021a). Increased SGK1.1 activity reduced the number and duration of systemic seizures in the hippocampus and cortex, leading to early termination of seizures. In addition, activation of SGK1.1 reduced the level of reactive glioma after persistent epilepsy and reduced apoptosis after Ka-induced epilepsy (Martin-Batista et al., 2021b), which undoubtedly provides two new ideas for the application of SGK1.1 in the treatment of epilepsy.

The above studies showed that SGK1 participates in different pathways and plays different roles in different nature of epilepsy. However, a large number of studies have shown that inhibiting the expression level of SGK1 is conducive to the treatment of epilepsy and SGK1 will be an important therapeutic target. Therefore, the molecular mechanism of SGK1 in epilepsy needs to be further explored.

### 3.4 SGK1 and post-traumatic stress disorder

Post-traumatic stress disorder (PTSD) is a complex disorder with many neurobiological alterations (Yehuda et al., 2015), which can be caused by fear, traumatic brain injury, etc. (Alves de Araujo Junior et al., 2023). PTSD is associated with changes in glucocorticoid sensitivity and gene expression in the human brain, and decreased Sgk1 gene expression levels have been found in the subgenus PFC and dorsolateral PFC, suggesting that glucocorticoid signaling is dysregulated in the brain (Duman and Girgenti, 2019). SGK1 is one of the most deregulated genes expressed in PTSD patients (Robinson, 2015), and it has been demonstrated in a rodent rat model that reduced SGK1 function in the prefrontal cortex of the rat leads to behaviors characteristic of traumatic stress, and inhibition of SGK1 expression using rAAV-dnSGK1, an adenovirus encapsulated to silence SGK1, significantly reduced the density of spines on dendrites of neurons in layer II/III, suggesting that SGK1 is required to maintain the structural integrity of dendritic spines in the PFC and to maintain normal brain function (Licznerski et al., 2015). Corticosteroid stress hormones have been found to have a strong effect on PFC function (Amaya et al., 2023; Yuen et al., 2011), with stress or short-term corticosterone treatment activating the glucocorticoid receptor to increase the transport and function of NMDAR and AMPAR via SGK/Rab4 signaling, leading to enhanced synaptic transmission, which in turn facilitates PFC-mediated cognitive processes (Yuen et al., 2011). Thus, in this pathway, it can be hypothesized that when SGK1 is blocked, Rab4 also loses its role, thus exacerbating cognitive impairment. Similarly, virus-mediated down-regulation of SGK1 in the medial PFC (mPFC) of rats revealed that reduction of SGK1 expression in the mPFC decreases resistance to stressful memory deficits (Park et al., 2021), suggesting that SGK1 is a key molecular and cellular mediator of cognitive impairment. Additionally, some of the cognitive deficits may be associated with increased tau protein deposition in the brain (Wei et al., 2019), specifically in SPS-induced PTSD rats, due to the incongruent interaction between AKT-related GSK-3β activation and SGK1-related ERK1/2 inactivation, which ultimately leads to hyperphosphorylation of tau proteins at the Ser202/Thr205 and Ser404 loci.

In conclusion, SGK1 plays an important role in the treatment of PTSD. Elucidating the upstream and downstream targets of SGK1 and the related signaling pathways may help to understand the molecular mechanism of cognitive impairment and provide new therapeutic targets for PTSD.

### 3.5 SGK1 and stroke

Stroke is a common neurological disorder that is a group of diseases in which brain tissue is damaged due to a sudden rupture of a blood vessel in the brain or a blockage in a blood vessel that prevents blood from flowing to the brain. One of the more important types of stroke classification is ischemic stroke, the cause of which is mainly induced by insufficient or untimely blood supply to the brain due to the formation of blood clots in the brain (Barthels and Das, 2020). Cerebral ischemia causes inflammation, oxidative stress and mitochondrial dysfunction, and microglia have been found to have an important role in cerebral ischemic inflammation (Kim and Cho, 2016). It has been found that inhibition of SGK1 activity exacerbates the inflammatory response of microglia (Inoue et al., 2016b), and dimethylamino tetracycline inhibits microglial cell activity in an animal model, which in turn attenuates the negative impacts caused by stroke (Yrjänheikki et al., 1999). Based on this finding, microglia hyperactivation can be controlled by stimulating the upregulation of Sgk1 gene expression without side effects (Asai et al., 2018). Cl^-^ spillover mediated by Swell1 (Cl^-^ transmembrane transporter protein) in microglia has been found to activate SGK1, which in turn achieves a significant reduction in ischemic brain injury through the FOXO3a/CREB signaling pathway(Chen et al., 2023). Therefore, it can be hypothesized that activating the expression of SGK1, which is partially associated with microglia, contributes to the treatment of ischemic stroke. Elsewhere, the protective effect exerted by a-phenyl-n-tert-butyl nitrone in stroke is mediated by rapid induction of SGK1 expression (McCaig et al., 2019). Intracarotid cold saline infusion increases the expression of SGK1 and decreases the expression of autophagy markers beclin-1 and LC-3 in a rat model of ischemic stroke, thereby creating a neuroprotective effect on stroke (Wang et al., 2019). In hypoxic-ischemic brain injury, short-chain fatty acids were validated to inhibit inflammation produced by cerebral ischemia by reducing astrocyte activation through the SGK1/IL-6 signaling pathway (Gao et al., 2022).

From the above, it seems that high SGK1 gene expression plays a therapeutic role in stroke, however, other studies have found that SGK1 expression plays a negative role in stroke treatment. In animal models, SGK1 inhibitors reduced NMDA receptor-mediated neurotoxicity, decreased Nedd4–2 phosphorylation and inhibited voltage-gated sodium currents, which led to a significant reduction in the volume of cerebral infarcts in the MCAO adult mouse model (Inoue et al., 2016a). Inhibition of SGK1 by the SGK1 inhibitor GSK650394 has been found to reduce infarct size and blood-brain barrier disruption in ischemia-reperfusion injury during the early phase of cerebral ischemia, thereby protecting brain function (Chi et al., 2021). In stroke, lesions of the blood–brain barrier and the formation of brain edema are often associated with each other, and both are important features of pathological changes in stroke. In high-salt diet fed mice, high salt indirectly disrupts the blood-brain barrier by decreasing the expression of blood-brain barrier tight junction proteins through the p38/MAPK-SGK1 signaling pathway, which further negatively affects cerebral ischemia (Zhang et al., 2015). Excessive sugar intake in a high-glucose environment exacerbates edema formation and worsens neurological prognosis in ischemic stroke (Klug et al., 2021), and the main reason is that the presence of high glucose increases the activity of SGK1, which in turn increases the abundance of Na^+^-K^+^-2Cl^-^ cotranslocator proteins and Na^+^/H^+^ exchange proteins, thus disrupting the water-salt balance of the blood-brain barrier and exacerbating brain edema.

In summary, the presence of SGK1 plays a double-edged role for ischemic stroke, as it attenuates neuroinflammation on the one hand, while on the other hand it plays a negative role in CA-induced cerebral ischemia and the blood-brain barrier changes in stroke. Therefore, the ultimate effect of SGK1 on stroke requires further exploration of the deeper mechanisms.

### 3.6 SGK1 and neuropathic pain

Neuropathic pain (NP) is a long-term recurrent disorder caused by damage to the somatosensory nervous system. The clinical manifestations are mainly spontaneous pain, nociceptive hypersensitivity, allodynia, and sensory abnormality, which brings a huge burden to people's lives (Li et al., 2022). SGK1 plays a role in the development of various types of pathologic pain, and targeting SGK1 may be a novel pain treatment strategy (Géranton et al., 2007). In recent years, a number of studies have shown that SGK1 plays a role in pathologic pain via spinal cord or spinal cord neurons. Phosphorylated SGK1 may interact with kalirin (one of the most highly phosphorylated targets in postsynaptic density (PSD), and enhance its expression, which in turn induces phosphorylation of the NR2B subunit associated with PSD-95-NR2B coupling in dorsal horn neurons, resulting in NP (Peng et al., 2013). In a rat model studying spinal nerve ligation, phosphorylation of SGK1 was found to accumulate in the nucleus, followed by HDAC4 phosphorylation in the nucleus of dorsal horn neurons, and the phosphorylated HDAC4 was coupled to the 14–3–3β protein (a phosphorylated nuclear protein), which increased the abundance of dorsal horn 14–3–3β proteins, which then exacerbated the neurologic injury and NP (Lin et al., 2015). Pain and inflammation often arise concomitantly, and SGK1 protein levels are increased in dorsal horn neurons after inflammation, and reduced SGK1 expression in neuron can delay the onset of pain (Géranton et al., 2007). NP is subject to a circadian rhythm, and in a mouse model of sciatic nerve injury, this circadian variation was found to be mediated by glucocorticoid-induced enhancement of ATP release from astrocytes through the pannexin-1 hemichannel (Koyanagi et al., 2016). SGK1 was found to enhance ATP release from spinal astrocytes in a SPS-induced rat model, corroborating the effect of the glucocorticosteroids-SGK1-ATP signaling pathway on NP (Zhang et al., 2019). Incidentally, other scholars have demonstrated that spinal expression of SGK1 is associated with glucocorticoid-induced exacerbation of NP hypersensitivity (Yasukochi et al., 2021), which is induced by SGK1-mediated stimulation of ATP production by glucocorticosteroids, and sulfasalazine inhibits SGK1 activity in spinal dorsal horn microglia and attenuates the pain hypersensitivity in peripheral nerve-injured mice. These studies all demonstrated that the most direct cause of NP is because SGK1 enhances the release of ATP from spinal cord astrocytes. In a skin/muscle incision and contraction rat model, it was demonstrated that nerve growth factor in the dorsal root ganglion may be involved in NP partly through the activation of SGK1 phosphorylation of Nedd4–2 to up-regulate the sodium channel Nav1.7 (Liu et al., 2021). In postoperative NP due to SMIR, both extracellular signal-regulated kinase phosphorylation and SGK1 phosphorylation are upregulated in the dorsal horn of the spinal cord, which is mitigated by injections of an SGK1 inhibitor (Li et al., 2023), implying that activation of phosphorylation of SGK1 leads to increased production of pro-inflammatory mediators.

Taken together, SGK1 plays a negative role in NP. SGK1 promotes the release of ATP to promote NP, and a series of downstream pathway signals induced by SGK1 phosphorylation aggravate painful inflammatory responses, suggesting that inhibition of SGK1 activity is a potential target for the treatment of NP.

### 3.7 SGK1 and major depressive disorder

Major depressive disorder (MDD) is a multifactorial disorder caused by environmental and genetic factors (Lupien et al., 2009). However, the pathogenesis at the genetic and molecular levels is not fully understood. In recent years, there have been many hypotheses to explain the pathogenesis of MDD, one of which is the stress disorder of the hypothalamic-pituitary-adrenal cortex (HPA) system, where glucocorticoid receptor (GR) plays an important role by regulating the signaling pathway mediated by downstream target gene SGK1. The increase of SGK1 expression during long-term stress decrease GR level, and SGK1 mRNA level is negatively correlated with GR level in clinical samples (Cattaneo and Riva, 2016). Sustained stress can cause a continuous increase in plasma corticosterone levels, induce PDK1 phosphorylation and up-regulate SGK1 mRNA expression in oligodendrocytes, leading to excessive differentiation of oligodendrocytes (Miyata et al., 2015). Specifically, chronic stress induces nuclear translocation of phospho-SGK1, which reduces oligodendrocyte activity by suppressing mGluR3 and 5 expression, and further destroys the tissue of Ranvier lymph nodes, leading to MDD related specific white matter abnormalities (Miyata et al., 2016).

In hippocampal astrocytes, electroconvulsive therapy plays a potential role in anti-depression. In a mouse model of depression, corticosterone-induced reduction in the number of hippocampal astrocytes was found to be caused by SGK1, and electroconvulsive therapy inhibited SGK1 expression and promoted the occurrence of antidepressant behavior (Miyako et al., 2024). However, another study showed that electroconvulsive therapy had no significant effect on GR and SGK1 mRNA levels in peripheral blood by analyzing whole blood samples before and after electroconvulsive therapy, suggesting electroconvulsive therapy can not play a good therapeutic role in patients with depression (Ryan et al., 2020). In addition, a study showed that the level of SGK1 mRNA in peripheral blood of depressed patients who did not receive medication was increased (Anacker et al., 2013), but the expression of SGK1 was reduced in MDD patients with small hippocampus (Frodl et al., 2012). At present, many studies have not yet perfected the exploration of the pathogenesis of SGK1's involvement in MDD, and there are many conflicting conclusions, such as whether the above electroconvulsive therapy is effective for MDD recovery, and whether SGK1 is elevated or decreased in MDD patients. Therefore, we cannot conclude whether inhibiting SGK1 or promoting it is conducive to the recovery of MDD, but it is undeniable that SGK1 participates in the important process of MDD pathogenesis and is a potential target for MDD treatment.

### 3.8 SGK1 and other neurologic disorders

Huntington's disease (HD) is a rare inherited neurodegenerative disorder that can lead to progressive movement disorders, psychiatric symptoms, and cognitive impairment. The underlying cause is a mutation in the Huntington gene, caused by a repeat of the elongated trinucleotide CAG (Fields et al., 2021). In recent years, mutated Huntington proteins have been implicated in various mechanisms by which they exert their deleterious effects in HD patients, including but not limited to NMDA receptor-mediated excitotoxicity, mitochondrial dysfunction and oxidative stress, dyshagy, and abnormal protein aggregation, of which excitotoxicity may be inextricably linked to SGK1 (Kim et al., 2021a). In 2001, selective degeneration of the γ-hydroxybutyrate medium spiny neuron population has been shown to be due to excitotoxicity mediated by NMDA receptor (Zeron et al., 2001), and on the basis of this finding it has been shown that striatal neurodegeneration in HD predominantly affects the γ-hydroxybutyrate spiny neuron population(Ehrlich, 2012). SGK1 inhibitors can reduce NMDA receptor-mediated neurotoxicity through SGK1 (Inoue et al., 2016a), and glucocorticoids can modulate NMDA receptor expression through activating SGK1 (Lengel et al., 2022). It is hypothesized that SGK1 inhibition may suppress the deleterious effects of mutant Huntington proteins by attenuating the excitotoxicity of NMDA.

Chorea acanthocytosis (ChAc) is a neurodegenerative disease in which the molecular basis of the lesion is the absence of the functional chorea protein vesicular protein sorting-associated protein 13 A (Ueno et al., 2001; Velayos-Baeza et al., 2004), which leads to apoptosis. It has been found that down-regulation of ORAI1 (calcium pore-forming channel protein) expression and decreased subunit of pore-forming calcium channel components are involved in the pathophysiology of ChAc. Neurons generated from fibroblasts of patients with ChAc through the induction of pluripotent stem cells express less ORAI1 and STIM1 proteins than neurons generated from healthy control fibroblasts, and the apoptosis of former neurons was enhanced (Lang et al., 2017, Pelzl et al., 2017). Since ORAI1 is a target of Nedd4–2, SGK1 inhibits neuronal apoptosis and slows ChAc by disrupting Nedd4–2-mediated ubiquitination of ORAI1 and upregulating NF-κB-sensitive ORAI1/STIM1 expression (Lang et al., 2018).

Multiple sclerosis (MS) is a central nervous system disease that affects 1 million people worldwide (Ebers, 2008). Oxidative stress plays an important role in the development of MS, and it is well known that stress activates the p38/MAPK-SGK1 signaling pathway, which cumulatively generates an inflammatory response (Ebers, 2008). A number of recent studies have identified MAPK activation as a central participant in MS and experimental autoimmune encephalomyelitis (EAE) (van der Meer and Netea, 2013). In an experimental EAE model (Pelletier and Hafler, 2012), increased expression of p38MAPK and SGK1, enhanced induction of Th17 cells, and elevated levels of oxidative stress exacerbated the clinical symptoms of EAE (Wang et al., 2017), implying that the p38MAPK-SGK1 pathway exerts a negative effect on the treatment of MS.

## 4 SGK1 inhibitor

In recent years, based on the findings that upregulation of SGK1 may negatively regulate neurological disorders, many scholars have carried out the role of SGK1 inhibition in the treatment of neurological disorders. In a tau transgenic mouse model, GSK650394 treatment was able to restore synaptic function of the PFC in tau transgenic mice compared to a control group injected with normal saline, thus aiding in the recovery of recognition and spatial memory in the mice (Cao et al., 2020). Injection of EMD638683 (one of the currently common SGK1 inhibitors) into SH-SY5Y cells inhibited time-dependent overexpression of tau without affecting normal tau levels, cell survival, or cytotoxicity (Elahi et al., 2021). Similarly, interfering with SGK1 synthesis by injecting GSK650394 or sh-SGK1-AAV9 into PD model mice revealed that both methods had a favorable modification of the symptoms of PD (Kwon et al., 2021). In ischemic stroke studies, injection of GSK650394 attenuates neuronal death and blood-brain barrier damage by ischemic stroke (Lee et al., 2020), and administration of AAV-SGK1-shRNA also alleviates neuroinflammation caused by stroke (Chi et al., 2021). Sulfapyridine inhibits SGK1 activity in spinal cord dorsal horn microglia with neuropathic pain and attenuates pain hypersensitivity in mice with peripheral nerve injury (Yasukochi et al., 2021).

In recent years, some novel SGK1 inhibitors have been discovered and are also being validated for the treatment of neurological disorders. A structure-based virtual high-throughput screening of natural compounds in the ZINC database identified the chemical structure of compound ZINC00319000 that efficiently binds to SGK1 to form stable complexes (Mohammad et al., 2020). Similarly, a novel SGK1 inhibitor, hit15, was screened from a database containing 29,158 compounds and showed high inhibition activity against SGK1, with a inhibition rate of 44.79 % at 10 µM concentration (Zhang et al., 2022). Based on the lack of ability of previous SGK1 inhibitors to cross the blood–brain barrier, through virtual screening and chemical structure analysis, it has been found that some molecules belonging to the deazapurine family can cross the blood-brain barrier to reach the central nervous system and protect neurons (Maestro et al., 2023). Taken together, the discovery of these novel SGK1 inhibitors may have a positive impact and therapeutic effects for diseases associated with SGK1 activation.

## 5 Conclusion and prospect

In this paper, we reviewed the role of SGK1 in various neurological diseases such as AD, PD and epilepsy, etc. (Fig. 2), and found that SGK1 may play a positive feedback, negative feedback, or dual roles according to its "role" in the signaling pathways involved in various diseases (Table1). However, since SGK1 plays a "double-edged sword" role in the development of some diseases, the brain regions and signaling pathways involved in the diseases need to be carefully evaluated if SGK1 is to be used as a drug target for the treatment of neurological diseases. As the study develops in depth, further elucidation of the molecular mechanism of SGK1 in various neurological diseases, as well as strengthening the development of biopharmaceuticals and molecular inhibitors targeting SGK1, can provide important theoretical basis and therapeutic means for the treatment of related clinical diseases.

**Table 1:** The pathophysiological role of SGK1 in various neurological disorders.

Neuropathic disease	Actions of SGK1	Functional outcomes	References
Alzheimer's disease	Accelerate Aβ degradation, γ-secretase phosphorylation,	Reduction of AD symptoms	Mo et al. (2012); Chow et al. (2015); Lian et al. (2020)
	Exacerbate tau protein phosphorylation	Aggravation of cognitive impairment	Elahi et al. (2021)
Parkinson's disease	Reduce α-syn levels	Reduction of PD-related symptoms	Heo et al., 2023, Seo et al., 2023, Yeo and Lim, 2019, Yeo et al., 2018
	Activate the NF-κB signaling pathway	Exacerbation of neuroinflammation	Kwon et al. (2021)
Epilepsy	Accelerate NEDD4–2 degradation	seizure aggravation	Kim et al. (2021b)
	Elevate glucose transporter protein levels and increase Lofora vesicle production	LD aggravation	Singh et al. (2013)
PTSD	Up-regulate activation of Rab4 protein	Reduction of cognitive impairment	Yuen et al. (2011)
Stroke	Activate the FOXO3a/CREB signaling pathway	Reduction of ischemic brain damage	Chen et al. (2023)
	Decrease the expression of beclin−1, LC−3	Protection of the cerebral nerves	Wang et al. (2019)
	Regulate Ndrg1-SOX10 axis	Aggravation of nerve damage	(Wu et al., 2024)
	Increase in Na + -K + −2Cl- cotransporter protein abundance	Destruction of the BBB	Zhang et al. (2015)
Neuropathic pain	Enhance spinal ATP release	Increasing neuropathic pain	Koyanagi et al. (2016); Zhang et al. (2019); Yasukochi et al. (2021)
	Mediate NEDD4–2 phosphorylation to upregulate Nav1.7		Liu et al. (2021)
	Enhance interaction with kalirin		Peng et al. (2013)
	Up-regulate HDAC4 phosphorylation		Lin et al. (2015)
Major depressive disorder	Regulate GR phosphorylation	Aggravate MDD	Anacker et al., 2013, Cattaneo and Riva, 2016
	Aggravate oligodendrocyte overdifferentiation		Miyata et al., 2015, Miyata et al., 2016
Huntington's disease	Regulate NMDA receptor expression	Striatalneuro- degeneration in HD	Ehrlich, 2012, Zeron et al., 2001
Chorea acanthocytosis	Up-regulates ORAI1/STIM1 expression	Inhibition of neuronal apoptosis	Lang et al. (2018)
Multiple sclerosis	Exacerbate oxidative stress	Exacerbation of MS disease	Wang et al. (2017)

AD: alzheimer's disease; PD: parkinson's disease; LD: lafora disease; PTSD: post-traumatic stress disorder; BBB: blood brain barrier; HD: huntington's disease; MS: multiple sclerosis.

MDD: major depressive disorder；GR: glucocorticoid receptor

## CRediT authorship contribution statement

**Yechen Xiao:** Writing – review & editing, Writing – original draft, Funding acquisition, Conceptualization. **Haixian Kang:** Writing – review & editing. **Xiuze Chen:** Writing – review & editing, Writing – original draft, Conceptualization.

## Declaration of Competing Interest

The authors declare that they have no known competing financial interests or personal relationships that could have appeared to influence the work reported in this paper.
